# Assessment of peripheral gene expression signatures as predictive biomarkers for hepatocellular carcinoma following DAA treatment

**DOI:** 10.1016/j.jgeb.2025.100583

**Published:** 2025-10-08

**Authors:** Rehab I. Moustafa, Sally Farouk, Noha G. Bader El Din, Hend I. Shousha, Ahmed Khairy, Yasser K. Elesnawy, Heba Shawky, Ahmed M. Gabr, Ashraf O. Abdelaziz, Amr Abdelaal, Hassan Elsayed

**Affiliations:** a Department of Microbial Biotechnology, Biotechnology Research Institute, National Research Centre, Dokki, Cairo 12622, Egypt; b Endemic Medicine and Hepato-Gastroenterology Department, Cairo University, Cairo, Egypt; c National Committee for Control of Viral Hepatitis (NCCVH), Ministry of Health and Population, Cairo, Egypt; d Therapeutic Chemistry Department, Pharmaceutical Industries and Drug Research Institute, National Research Centre, Dokki, Cairo 12622, Egypt; e Department of Surgery, Faculty of Medicine, Modern University for Technology and Information, Cairo, Egypt; f Department of Surgery, Faculty of Medicine, Ain Shams University, Cairo, Egypt

**Keywords:** HCC, HCV, SVR, PBMCs, JUNB, WNT10A, SPHK1, EDN1, KLF4

## Abstract

Hepatocellular carcinoma (HCC) is a major cause of cancer mortality worldwide, with viral hepatitis accounting for about 80 % of incidences. In Egypt, HCV contributes to 63 % of HCC cases. Although DAAs have achieved high SVR rates, they do not eliminate the risk of HCV-related HCC. Persistent epigenetic changes induced by HCV-infection may establish an “oncogenic memory” that promotes HCC even after viral clearance. Peripheral blood mononuclear cells (PBMCs) offer a non-invasive platform for detecting systemic immune and oncogenic signatures, aiding HCC risk assessment. This study aimed to characterize the expression of an epigenetically induced gene panel, comprising JUNB, WNT10A, SPHK1, EDN1, and KLF4 in hepatic tissues and PBMCs from Egyptian HCC patients with HCV genotype 4 who achieved SVR. In silico analyses revealed strong epigenetic associations of these genes, including links to histone-modifying enzymes, protein–protein interaction networks, and enrichment in cancer-related pathways. Gene expression was analyzed using qRT-PCR in SVR individuals, chronic HCV patients, and healthy controls, with diagnostic performance evaluated using multivariate regression and ROC curve analyses. Our results showed significant upregulation of WNT10A, SPHK1, JUNB, and EDN1 and downregulation of KLF4 in PBMCs, particularly post-SVR. PBMC expression showed high diagnostic accuracy (AUROC > 0.92 for SPHK1, WNT10A, JUNB). In conclusion, combining PBMC gene expression profiling with in-silico analyses highlights JUNB, WNT10A, SPHK1, EDN1, and KLF4 as promising non-invasive biomarker panel for HCC risk in DAA-SVR patients, reflecting their integration into epigenetic and oncogenic networks and supporting their potential for risk stratification and therapeutic targeting.

## 1 Introduction

Hepatocellular carcinoma (HCC) ranks as the sixth most common cancer and the third leading cause of cancer-related mortality worldwide.^1^, ^2^ HCC has been attributed to major risk factors including liver cirrhosis, alcohol abuse, metabolic-linked fatty liver disease, and viral hepatitis, with chronic hepatitis C (HCV) infections and chronic hepatitis B (HBV) accounting for around 80 % of global cases.^3^ HCV, infecting around 50 million people globally, establishes chronicity in approximately 70 % of infected individuals. Around 20 % of long-lasting chronic HCV infections develop liver cirrhosis, from which around 4 % will develop HCC yearly.^4^

Currently, direct-acting antivirals (DAAs) are the standard treatment for HCV. While DAAs have improved the HCV eradication rates, increased the sustained virological response (SVR), they do not eliminate HCC risk, particularly in patients with advanced fibrosis or cirrhosis.^5^, ^6^, ^7^, ^8^, ^9^, ^10^, ^11^, ^12^ In Egypt, which is a high-prevalence region for HCV, particularly genotype 4, the widespread DAA treatment has reduced HCV prevalence and mortality, meeting WHO elimination targets.^13^, ^14^, ^15^ This achievement represents one of the largest public health efforts aimed at eliminating hepatitis C in middle- and low-income countries.^16^ However, HCC remains a leading cause of cancer mortality in Egyptian SVR populations, with discrepancies in post-SVR HCC incidence highlighting the need for biomarkers to identify at-risk patients, particularly for genotype 4-driven HCC.^15^, ^17^, ^18^, ^19^

HCV, a cytoplasmic virus with low integration potential into the host genome, drives hepatocarcinogenesis through both direct and indirect pathways. Indirectly, chronic inflammation, steatosis, liver fibrosis, and cirrhosis create an oncogenic microenvironment in the liver, while direct effects involve HCV proteins, which disrupt cell signaling, apoptosis, and angiogenesis.^20^, ^21^ HCV influences these processes by altering numerous genetic and epigenetic factors in host cells, thereby contributing to HCC carcinogenesis.^6^

Transcriptomic and epigenomic studies have demonstrated that HCV infection leaves behind lasting epigenetic alterations inducing transcriptional dysregulation that persists post-SVR, thereby contributing to HCC progression.^22^, ^23^, ^24^ This persistent aberrant gene expression, observed in HCV-infected liver tissues, suggest an “oncogenic memory” that contributes to HCC risk even after viral clearance.^25^, ^26^

Unlike traditional serum or plasma biomarkers, which exhibit variability due to multi-tissue origins, Peripheral blood mononuclear cells (PBMCs) offer a more consistent and reliable source for biomarker discovery. Their single-cell origin allows them to accurately reflect systemic immune status and tumor microenvironment dynamics, including immune evasion and cancer immunogenicity.^27^ Studies indicate that changes in gene expression in PBMCs may correlate with tumor immunogenicity and the host's ability to combat cancer.^28^, ^29^, ^30^ For instance, the presence of specific immune cell clones in PBMCs is associated with enhanced anti-tumor activity, suggesting that these cells can mirror the dynamics occurring within the tumor microenvironment.^31^ By offering a non-invasive alternative to liver biopsies and addressing the challenge of intratumor heterogeneity, PBMCs support the development of diagnostic and prognostic tools for HCV-induced HCC. They also hold promise for personalized medicine through the identification of molecular signatures, immune checkpoint pathways, and tumor-associated genes.^32^, ^33^, ^34^

This study focused on the epigenetically induced “transcriptional fingerprint” left by HCV, exploring persistent gene expression changes in Egyptian patients with HCV-related HCC, particularly post-SVR. To that end, we focused on five genes, comprising WNT10A, SPHK1, JUNB, EDN1, and KLF4, which have been reported to undergo epigenetic alterations in HCV infection,^23^, ^24^ and complemented this with in silico analyses, which revealed links to histone-modifying enzymes, protein–protein interaction networks, and enriched pathways, highlighting their biological relevance. We aimed to characterize the expression of these genes in both hepatic tissues and PBMCs from Egyptian patients with HCV genotype 4–related HCC, either post-DAA treatment or untreated, as well as in SVR patients without HCC. Expression profiles were further compared with those of chronic HCV patients and healthy controls. While prior studies have examined the expression of these genes in hepatic tissues of HCV-infected and SVR patients we sought to determine whether similar dysregulation occurs in PBMCs, which could offer a non-invasive approach for early HCC detection. By elucidating the HCV-induced oncogenic memory, we aimed to identify novel biomarkers for HCC risk assessment and prognosis, as well as potential therapeutic targets, to enable precision interventions and reduce the burden of HCC in Egypt.

## 2 Materials and methods

### 2.1 Study design

**Fig. 1:**
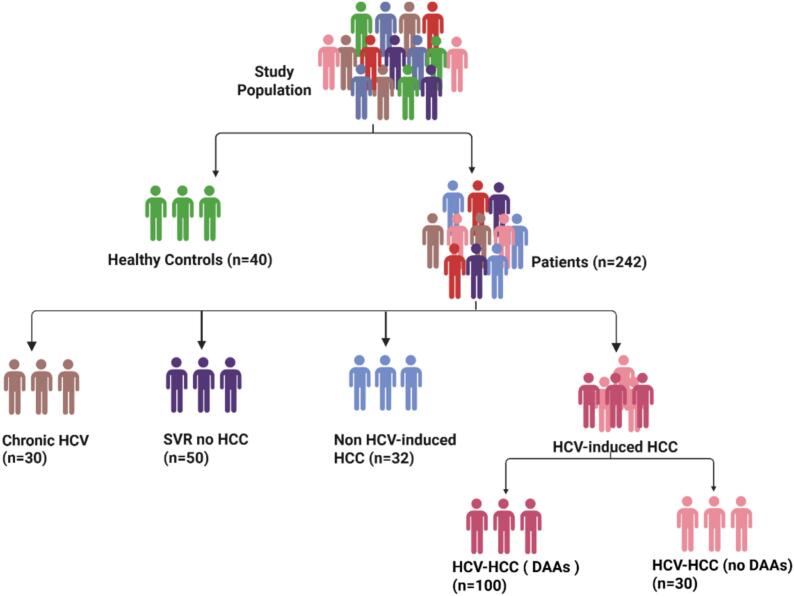
Schematic representation of the study design for PBMC gene expression analysis.

This two-phase study investigates the persistent molecular footprint of hepatitis C virus (HCV) infection after sustained virologic response (SVR) and its potential as a prognostic indicator for hepatocellular carcinoma (HCC). The study evaluates differential expression of five candidate genes (WNT10A, JUNB, SPHK1, EDN1, and KLF4) to identify HCV-associated molecular signatures. In Phase I, hepatic gene expression is analyzed in liver biopsies to detect patterns indicative of a post-HCV molecular signature linked to HCC risk. In Phase II, gene expression analysis is extended to peripheral blood mononuclear cells (PBMCs) from an expanded cohort to explore these genes as non-invasive biomarkers for early identification of high-risk HCC patients, as depicted in Fig. 1.

### 2.2 Human subjects

Liver tissues and their corresponding peripheral blood samples were collected from patients at the Liver Transplant Department, Air Force Specialized Hospital (Cairo, Egypt). Eligible patients were selected through systematic medical record review. Liver biopsies from HCV-related cases were graded and staged using the METAVIR classification system at the pathology department of the Air Force Specialized Hospital.

In Phase I, we assessed hepatic gene expression using qRT-PCR in liver biopsies from four distinct groups. Group I: (Healthy controls) Individuals with no history of liver disease, confirmed by normal hematological and biochemical parameters (n = 10), Group II: (HCV-HCC with DAAs) Patients with HCV-related HCC who developed cancer after achieving SVR via direct-acting antivirals (DAAs) (n = 9), Group III: (HCV-HCC (no DAAs)) Patients with HCV-related HCC patients, who did not receive DAA treatment (n = 7), and Group IV: (Non-HCV-HCC patients) Patients with HCC due to other etiologies (e.g., HBV, nonalcoholic steatohepatitis [NASH])(n = 11). Expression levels were normalized to healthy individuals to identify patterns indicative of a persistent HCV-associated molecular signature.

For gene expression validation in PBMCs (Phase II), additional subjects were recruited at the Multidisciplinary HCC clinic, Kasr Al-Ainy Teaching Hospital, Faculty of Medicine, Cairo University, to achieve the following group sizes. Group I: Healthy Controls (n = 40); Individuals with no history of liver disease, confirmed by normal clinical parameters, serving as a reference for gene expression analysis. Group II: HCV-HCC with DAAs (n = 100), Group III: HCV-HCC (no DAAs) (n = 30), Group IV: Non-HCV-HCC (n = 32). In Addition to Group V: Chronic HCV (no HCC) (n = 30); Patients with chronic HCV infection, untreated with DAAs, without HCC development, and Group VI: SVR without HCC (n = 50), Patients who achieved SVR via DAA therapy at least two years prior, without HCC development. Peripheral blood samples (5 mL) were collected in EDTA anticoagulant vacuum tubes from each participant.

### 2.3 Inclusion and exclusion criteria

Patients were enrolled in the study according to the following inclusion criteria: chronic hepatitis C virus patients who did not receive any antiviral treatment by the time of enrollment and did not develop malignancy. Patients who achieved SVR after DAA treatment without developing HCC, and others who developed HCC after achieving SVR. This subgroup excluded patients with any other co-infection, such as HBV, HEV, or HIV, as well as those with a history of alcohol dependence or recreational drug use. A third group includes HCC patients due to any other etiological factors. HCC patients included individuals either treated with resection or systemic treatment.

Exclusion criteria included major comorbidities: cardiac, respiratory, renal failure, and patients with other known malignancies. Patients with uncontrollable systemic infections such as miliary Tuberculosis, HIV, and disseminated sepsis or psychiatric illness, as well as fibrolamellar type of HCC, were also excluded. All healthy controls had no evident diseases, disorders, or infections. They tested negative for HCV antibodies, HCV RNA, HBV DNA, HBsAg, and HB core antibodies, and showed no signs of liver involvement based on abdominal ultrasound findings. Moreover, none had a history of liver damage from drugs, metabolic conditions, or viral exposure. Additionally, Liver tissue samples representing healthy controls were collected from individuals donating liver segments for transplantation, with no evidence of liver pathology.

### Ethical statement

All research procedures involving human participants were conducted in accordance with the ethical guidelines established by the institutional and/or national research committees, as well as the 1964 Declaration of Helsinki and its subsequent amendments or equivalent ethical guidelines. The study protocol received formal approval from the Ethics Committee of the National Research Centre (Approval Number # 20113). Furthermore, written informed consent was obtained from all participants prior to their enrollment in the study.

### 2.5 RNA extraction from tissue and blood samples

Tissue samples were immediately preserved in Allprotect Tissue Reagent (Qiagen, Hilden, Germany) and stored at –70 °C. Liver tissues were lysed in Buffer RLT (lysis buffer) and homogenized using Qiagen Tissue Lyser LT (Qiagen, Hilden, Germany). RNA was then purified from tissues using AllPrep DNA/RNA/Protein Mini Kit (Qiagen, Hilden, Germany) according to the manufacturer’s instructions. For Total RNA extraction from PBMCs, the QIAamp® RNA Blood Mini Kit (#52304; Qiagen, Hilden, Germany) was used for extraction from whole blood. Then, RNA quantity and quality were assessed using NanoDrop 2000c Spectrophotometer (Thermo Scientific, A.B., Missouri City, TX, USA).

### 2.6 Real-time PCR and gene expression assay

To generate cDNA, equal amounts of the RNA were then reversed transcribed using the QuantiTect Reverse Transcription Kit (#205311Qiagen, Hilden, Germany). Briefly, for efficient removal of any genomic DNA, RNA was mixed with gDNA Wipeout buffer and RNase-free water and incubated at 45 °C for 8 min. Following genomic DNA elimination, the RNA sample is reverse transcribed by adding Quantiscript Reverse Transcriptase enzyme, RT buffer, and RT primer mix. The reaction mixture was then incubated at 25 °C for 3 min, 45 °C for 20 min, followed by 85 °C for 5 min.

Relative mRNA expression levels were determined by qualitative real-time PCR using QuantiNova SYBR Green PCR Kit (#208052; Qiagen, Hilden, Germany). Briefly, the cDNA template was mixed with 2x QuantiTect SYBR–Green Master Mix, commercial QuantiTect SYBR Primers (#249900|; Qiagen, Hilden, Germany) listed in Table 1. and RNase–free water. The reaction was initiated with an initial activation step at 95˚C for 2 min, followed by 40 cycles of amplification, each consisting of denaturation at 95˚C for 5 sec, annealing at 60˚C for 10 sec, and extension at 70˚C for 30 sec. The reaction was performed on the Rotor–Gene PCR cycler (Qiagen, Hilden, Germany). Data were analyzed using the ΔΔCT method, with the housekeeping gene (*ACTB*) as normalizer.^35^

**Table 1:** List of Primers.

Gene symbol	Gene name	GeneGlobe ID#	Detected transcript
WNT10A	Homo sapiens Wnt family member 10A	QT00205632	NM_025216
JUNB	Homo sapiens Jun B proto-oncogene, Homo sapiens AP-1 transcription factor subunit	QT00201341	NM_002229
SPHK1	Homo sapiens sphingosine kinase 1	QT01011927	NM_001142601
KLF4	Homo sapiens Kruppel-like factor 4	QT00061033	NM_004235
EDN1	Homo sapiens endothelin 1	QT00088235	NM_001955
ACTB	Homo sapiens actin beta	QT00095431	NM_001101

### 2.7 In-silico analyses

Correlations between histone-modifying enzymes and dysregulated gene mRNA expression in HCC were assessed using the GEPIA2 correlation analysis module with the TCGA-LIHC dataset (http://gepia2.cancer-pku.cn/#index). Correlation coefficients (R) and P-values were obtained and visualized with Srplot.^36^ Protein–protein interactions of the studied genes were analyzed using the PINA v3 network construction module with cancer type specified as TCGA-LIHC. The resulting protein list was then uploaded to ShinyGO 0.85 for Gene Ontology (GO) and Kyoto Encyclopedia of Genes and Genomes (KEGG) pathway enrichment visualization.

### 2.8 Statistical analysis

Data analysis was conducted using GraphPad Prism version 9.1.2. Continuous variables are presented as mean ± standard deviation (SD) or as median (range) ± standard error (SE), while categorical variables are presented as frequencies and percentages. Comparisons among multiple groups were performed using one-way ANOVA with Tukey’s post hoc test for parametric data, or Kruskal-Wallis with Dunn’s test for non-parametric data. Multivariate logistic regression was performed to identify independent predictors of hepatocellular carcinoma development and to control for potential confounders. Results are expressed as odds ratios (ORs) with 95 % confidence intervals (CIs). The diagnostic performance of selected biomarkers was evaluated using receiver operating characteristic (ROC) curve analysis, including sensitivity, specificity, positive predictive value (PPV), negative predictive value (NPV), and overall accuracy, all reported with 95 % CIs. The identified cutoff values were further confirmed using Fisher’s exact test. All statistical analyses were two-tailed, with significance set at P ≤ 0.05.

## 3 Results

### 3.1 General characteristics of patients

#### 3.1.1 Demographic and clinical characteristics of the study groups

Demographic and clinical data for different study groups are summarized in Table 2. All HCC groups had a significantly higher median age than healthy controls (43 years, P < 0.0001), whereas no age difference was observed between chronic HCV and healthy individuals. To account for age-related effects on gene expression, age was included as a covariate in the Multivariate logistic regression analysis. Moreover, a significant male predominance was observed among HCC patients, compared to controls (P < 0.0001). The healthy control group exhibited no liver dysfunction, diabetes mellitus, or other diagnosed conditions, with hematological and biochemical parameters within normal ranges, confirming their stable health status and their suitability as a reference group for gene expression normalization. In contrast, HCC patients had elevated ALT, AST, AFP, and Child-Pugh scores (B7–B9), indicating significant liver impairment. Whereas chronic HCV and SVR patients had lower AFP and Child-Pugh scores, reflecting milder liver damage. Additionally, all HCC patients showed anemia and thrombocytopenia.

**Table 2:** Demographic and Clinical Characteristics of Study Cohorts.

Variable	Control (n = 40)	Patients (n = 242)					
		**Non HCV-induced HCC (n = 32)**	**HCV-induced HCC** **(n = 130)**		**HCV (n = 80)**		**P-value**
			**DAA** **(n = 100)**	**No DAA** **(n = 30)**	**Chronic** **(n = 30)**	**SVR** **(n = 50)**	
Gender (% [n/total])							
Male	60 (24/40)	84.37 (27/32)	73 (73/100)	83.34 (25/30)	66.67 (20/30)	48 (24/50)	<0.0001
Female	40 (16/40)	15.62 (5/32)	27 (27/100)	16.67 (5/30)	33.34 (10/30)	52 (26/50)	
Age (Years)	43 (19 – 63) ± 1.931	57 (30 – 71) ± 1.505	58 (42 – 75) ± 1.044	57 (44 – 71) ± 1.22	39.5 (22 – 85) ± 1.958	55 (28 – 72) ± 1.092	<0.0001
Clinical Assessment (% [n/total])							
Hypertension	−	46.8 (15/32)	31 (31/100)	43.34 (13/30)	10 (3/30)	56 (28/50)	0.0001
DM		31.25 (10/32)	41 (41/100)	33.34 (10/30)	6.67 (2/30)	44 (22/50)	0.018
Hematological profile & Blood Biochemistry							
Hb (g/dL)	13.8 (11.5–17.1) ± 0.221	12.4 (8.3 –16.7) ± 0.367	12.3 (7.3– 17.3) ± 0.376	11.0 (8.2 – 17.9) ± 0.378	13.3 (10 – 17) ± 0.248	12.4 (9.5 – 16.6) ± 0.226	<0.0001
WBCs (×10^3^/µL)	6.9 (4.3 – 11.04) ± 0.4743	3.9 (2.3 –8.1) ± 0.4231	5.3 (1.6 – 13.4) ± 0.4272	6.0 (2.0 – 9.4) ± 0.3215	6.05 (3.4 – 24) ± 0.412	6.44 (3.9 – 9.5) ± 0.2978	0.001
PLT (×10^3^/ µL)	286.0 (164– 393) ± 11.97	94.0 (28– 315) ± 15.11	150.0 (44 –412) ± 13.73	150.0 (40 –303) ± 10.14	222.5 (80 – 403) ± 10.34	205 (129 –268) ± 4.152	<0.0001
INR	1.0 (0.93 – 1.41) ± 0.0152	1.5 (1.1 – 1.8) ± 0.0353	1.17 (0.8– 1.99) ± 0.029	1.155 (1.0 – 1.7) ± 0.0368	1.03 (0.5 –2.6) ± 0.03235	1.241 (0.912– 1.69) ± 0.01	<0.0001
ALT (IU/L)	16 (7 – 34) ± 1.02	32 (11– 69) ± 2.175	29 (14– 212) ± 4.097	40 (13– 139) ± 4.749	48 (11– 198) ± 5.43	26 (7.5 – 49) ± 0.8738	<0.0001
AST (IU/L)	18 (11 – 37) ± 1.032	61 (37 – 81) ± 2.198	40 (17 – 297) ± 6.916	40 (22 – 187) ± 6.978	37 (17 –144) ± 3.664	27 (9 – 49) ± 1.04	<0.0001
T. BiL (mg/dL)	0.4 (0.21 – 2.4) ± 0.0656	2.45 (0.36 – 4.9) ± 0.2261	0.96 (0.25 – 13.1) ± 0.2993	1.0 (0.33–4.2) ± 0.1444	0.6 (0.23 – 2.0) ± 0.041	0.525 (0.1– 0.79) ± 0.0146	<0.0001
S. Cr (mg/dL)	0.74 (0.43 – 1.34) ± 0.0288	0.8 (0.39 – 1.53) ± 0.0329	0.95 (0.4 – 3.3) ± 0.0552	1.0 (0.59–1.43) ± 0.0377	0.86 (0.6 – 1.3) ± 0.0242	0.6 (0.39– 0.8) ± 0.0164	<0.0001
Log AFP (ng/mL)	−0.13 (−0.37 – 0.13) ± 0.01664	0.64 (0.28– 4.25) ± 0.1392	1.04 (0.26 – 4.78) ± 0.1462	1.0 (−0.15 – 5) ± 0.2262	0.48 (−0.3– 1.04) ± 0.0595	0.275 (−0.16 – 0.77) ± 0.086	<0.0001
ALB (g/dL)	4.7 (4.1 –5.4) ± 0.0517	3.2 (2.26–4.3) ± 0.1001	3.65 (2.5 –4.9) ± 0.0696	3.15 (2.5 – 5.0) ± 0.1002	4.3 (3.7 –5.2) ± 0.0518	4.65 (3.6–6.24) ± 0.0578	<0.0001
Treatment	−	**−**	DAAs	No DAAs	−	DAAs	NA
Log_10_ viral load (IU/mL)	−	**−**	SVR	6.05 (5.7––6.5) ± 0.29	5.577 (4.24 –7.74) ± 0.0977	SVR	NA
Child-Pugh class & score							
A5-6	−	25 (8/32)	23 (23/100)	23.33 (7/30)	100 (30/30)	100 (50/50)	0.0003
B7-9		37.5 (12/32)	65 (65/100)	53.34 (16/30)	0.0 (0/30)	0.0 (0/50)	
C10-15		37.5 (12/32)	12 (12/100)	23.33 (7/30)	0.0 (0/30)	0.0 (0/50)	

Gender, Diabetes Mellitus (DM), Hypertension, and Child-Pugh class & score are presented as % (n/total). Hematological and biochemical parameters, including hemoglobin (Hb, g/dL), platelet count (PLT, ×10^3^/µL), alanine aminotransferase (ALT, U/L), total bilirubin (T. Bil, mg/dL), and log-transformed alpha-fetoprotein (Log AFP, ng/mL), are reported as mean (range). Statistical significance is indicated as: * *p < 0.05; ** p < 0.01; *** p < 0.001; **** p < 0.0001; ns, not significant.

### 3.2 Differential gene expression associated with HCV infection in liver tissue

**Fig. 2:**
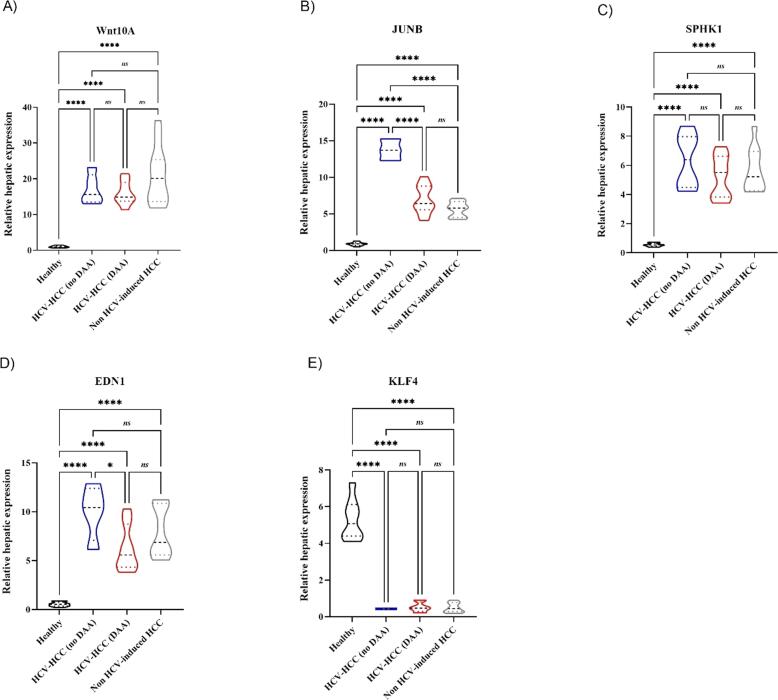
Relative hepatic gene expression in liver biopsies of HCC patients. (A–E) Violin plots illustrating the fold change in hepatic expression of (A) *Wnt10A*, (B) *JUNB*, (C) *SPHK1*, (D) *EDN1*, and (E) *KLF4* across four groups: healthy controls (Healthy), HCV-induced HCC patients without DAA treatment (HCV+/HCC), HCV-induced HCC patients treated with direct-acting antivirals (HCV−/HCC), and non-HCV-induced HCC patients. Expression levels were measured by qRT-PCR, and data are presented as fold change relative to healthy controls. Statistical significance was assessed using one-way ANOVA followed by Tukey’s post-hoc test. *p < 0.05, **p < 0.01, ***p < 0.001, ****p < 0.0001; ns, not significant.

To assess the differential gene expression in liver tissue of our study cohort, which consists entirely of Egyptian HCV genotype 4 patients, the predominant genotype in Egypt, we investigated gene expression changes associated with HCV. Using RT-qPCR, we analyzed a panel of the 5 selected genes (WNT10A, JUNB, SPHK1, EDN1, and KLF4), previously reported to be linked to HCV epigenetic modifications. The mRNA expressions of Wnt10A, JUNB, SPHK1, and EDN1 in HCC tissues were significantly upregulated in all HCC groups compared to the healthy group, while KLF4 was markedly downregulated, as shown in Fig. 2. The fold changes of the genes in different study groups are summarized in Table 3. Interestingly, the expression of JUNB was markedly elevated in all HCC groups, with a significant difference between the HCV-induced HCC receiving no DAAs, when compared to non-HCV-induced HCC groups and HCV-induced HCC with DAA. Moreover, the expression of EDN1 was significantly lower in the HCV-induced HCC group following viral cure in comparison to the untreated HCV-induced HCC group (p < 0.05).

**Table 3:** Mean fold change of differentially expressed genes in HCC tissue.

Differentially expressed genes	Log2 fold change (log2FC)		
	HCV-HCC (no DAAs) vs Healthy	HCV-HCC (DAAs) vs Healthy	Non-HCV-induced HCC vs Healthy
WNT10A	16.65	15.68	20.64
JUNB	15.91	8.03	6.52
SPHK1	11.64	9.55	10.42
EDN1	18.49	11.59	14.73
KLF4	0.08	0.10	0.09

### 3.3 Investigating gene expression profiles in the peripheral blood of different patient groups

Our findings demonstrate that Wnt10A, JUNB, SPHK1, EDN1, and KLF4 are differentially dysregulated in liver tissues of HCC patients, particularly those with HCV-induced HCC. These results support the potential of those genes as prognostic markers for HCC development. So, to assess their potential as non-invasive biomarkers, we analyzed their expression in peripheral blood mononuclear cells (PBMCs) from 242 individuals, including healthy controls, untreated chronic HCV patients, HCV-induced HCC patients (with and without DAA treatment), SVR individuals, and non-HCV-induced HCC patients.

**Fig. 3:**
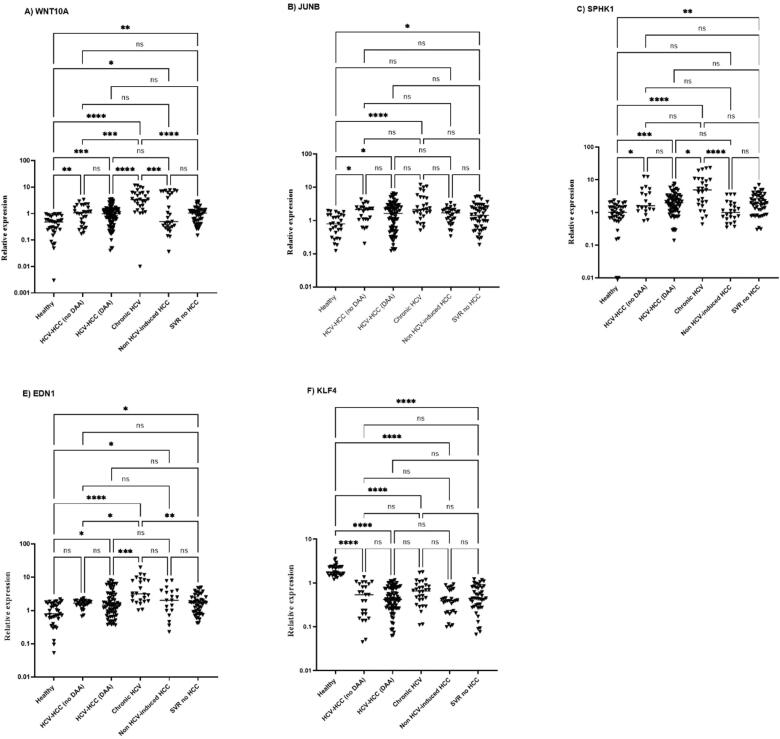
Relative gene expression of PBMCs across different patient groups Quantitative RT-PCR was performed to assess gene expression levels in healthy controls, HCV-induced HCC patients without DAA treatment, HCV-induced HCC patients with DAA treatment, chronic HCV patients, non-HCV-induced HCC patients, and sustained virologic response (SVR) patients. Data is presented as relative expression (fold change). Statistical significance was determined using the Kruskal-Wallis test followed by Dunn’s test, with p-values indicated as follows: p < 0.05 (*), p < 0.01 (**), p < 0.001 (***), p < 0.0001 (****), and “ns” denotes non-significant differences. Horizontal bars represent median values for each group.

The gene expressions of WNT10A, JUNB, SPHK1, and EDN1, as presented in Table 4, were significantly upregulated in chronic HCV patients (p < 0.0001) who had not received antiviral therapy yet, showing the highest expression among all groups. This upregulation persisted in HCV-induced HCC and SVR patients. When comparing SVR patients with HCV-induced HCC with DAAs, we observed slight differences in expression levels of certain genes; however, these variations were weak and did not reach statistical significance, as shown in Fig. 3. In contrast, non HCV-HCC patients showed no significant change in JUNB and SPHK1 expression, and only modest increases in WNT10A and EDN1 (p < 0.05). KLF4 was consistently downregulated across all HCC groups and in untreated chronic HCV patients (p < 0.0001), supporting its potential tumor-suppressive role.

**Table 4:** Median values and fold changes of differentially expressed genes in PBMCs.

Median with IQR						
Gene	Healthy	HCV-HCC (no DAA)	HCV-HCC (DAA)	Chronic HCV	Non HCV-induced HCC	SVR no HCC
WNT10A	0.47(0.24–0.81)	1.07(0.42–2.00)	0.98(0.46–1.46)	3.41(1.65–6.14)	0.5 (0.30–6.12)	1.04(0.54–1.32)
JUNB	0.79(0.39–1.47)	2.14 (1.06–2.49)	1.63(0.49–2.87)	2.16 (1.39–5.01)	1.70 (0.99–2.12)	1.42(0.66–2.54)
SPHK1	1.01(0.66–1.51)	1.60 (1.14–4.91)	1.97(1.13–3.05)	4.89(1.73–10.63)	1.00 (0.64–1.79)	1.93(0.95–3.19)
EDN1	0.79(0.57–1.62)	1.65 (1.37–1.95)	1.45(0.82–3.10)	3.15(1.91–8.12)	2.01(0.98–3.97)	1.71(0.90–2.51)
KLF4	1.77(1.53–2.34)	0.54 (0.17–0.94)	0.42 (0.26–0.74)	0.65(0.38–0.90)	0.39 (0.21–0.67)	0.44(0.28–0.79)
Fold change						
	Gene	HCV-HCC (no DAA)	HCV-HCC (DAA)	Chronic HCV	Non HCV-induced HCC	SVR no HCC
	WNT10A	2.23	2.04	7.11	1.04	2.17
	JUNB	2.69	2.06	2.72	2.14	1.79
	SPHK1	1.59	1.95	4.84	0.99	1.91
	EDN1	2.08	1.83	3.96	2.53	2.15
	KLF4	0.31	0.24	0.37	0.22	0.25

### 3.4 Discriminatory power of peripheral blood biomarkers

To comprehensively assess the prognostic factors for HCC, we performed a multivariate logistic regression analysis incorporating both baseline demographic and clinical data and our potential peripheral gene markers. The results, detailed in Table 5, indicate a strong baseline predictive capacity (6.295, OR = 542.1). Significant risk factors associated with increased HCC risk included Child-Pugh score, AFP, total bilirubin, lower albumin, and INR (P = 0.0047, P = 0.0007, P = 0.033, P = 0.0219, P = 0.0097, respectively). HCV infection significantly increases HCC risk (OR = 3.292), while DAA treatment showed a strong protective effect (OR = 0.0343) likely mitigating post-HCV HCC risk. Importantly, the peripheral mRNA fold changes of all five genes, Wnt10A, JUNB, SPHK1, EDN1, and KLF4, were evaluated and showed significant correlation with HCC. KLF4 showed the strongest inverse association (P < 0.0001, OR = 0.0047), followed by WNT10A (P = 0.0019, OR = 0.5069), EDN1 (P = 0.0124, OR = 0.7535), and SPHK1(P = 0.0356, OR = 0.719. JUNB, although positively associated, had a lower predictive value (*P* = 0.0281, OR = 1.407). These findings suggest that peripheral expression of these genes, especially KLF4, could serve as reliable non-invasive biomarkers for HCC risk stratification.

**Table 5:** Multivariate Logistic Regression Analysis of Clinical and Molecular Predictors for HCC Prognosis.

**Variable**	**Estimate**	**SE**	**95 % CI**	**t-ratio (Z)**	***P*-value**	**OR (95 % CI)**
**Intercept**	**6.295**	**1.357**	**3.95 to 9.38**	**4.64**	**< 0.0001**	**542.1 (51.94 to 11846)**
**Gender: Female (*Referent = Male*)**	**0.3059**	**1.836**	**−3.339 to 4.264**	**0.1666**	**0.8677**	**1.358 (0.03546 to 71.06)**
**Age (Years)**	**−0.013**	**0.051**	**−0.1288 to 0.081**	**0.2555**	**0.7983**	**0.9871 (0.8791 to 1.084)**
**HCV (*Referent = no*)**	**1.191**	**0.4792**	**0.2517 to 2.143**	**2.486**	**0.0129**	**3.292 (1.286 to 8.524)**
**Child-Pugh score**	**8.067**	**3.014**	**3.927 to 16.78**	**2.676**	**0.0047**	**3187 (50.75 to 2E + 07)**
**DAA (*Referent = Yes*)**	**−3.373**	**0.7057**	**−4.941 to −2.113**	**4.779**	**< 0.0001**	**0.0343 (0.0072 to 0.1208)**
**AFP (ng/mL)**	**2.84**	**0.8396**	**1.429 to 4.729**	**3.383**	**0.0007**	**17.11 (4.176 to 113.2)**
**ALT (IU/L)**	**−0.0299**	**0.0218**	**−0.081 to 0.0044**	**1.37**	**0.1706**	**0.9706 (0.9222 to 1.004)**
**AST (IU/L)**	**0.0249**	**0.027**	**−0.0173 to 0.088**	**0.9252**	**0.3548**	**1.025 (0.9828 to 1.092)**
**Total BiL (mg/dL)**	**0.906**	**0.4251**	**0.1481 to 1.938**	**2.132**	**0.033**	**2.475 (1.16 to 6.942)**
**ALB (g/dL)**	**−6.96**	**3.037**	**−14.85 to −2.316**	**2.292**	**0.0219**	**0.001 (3.57E-07 to 0.0987)**
**INR**	**13.09**	**9.737**	**−2.913 to 38.52**	**2.587**	**0.0097**	**1706 (14.64 to 9E + 05)**
**Peripheral *Wnt10A* (*mRNA fold change*)**	**−0.6795**	**0.219**	**−1.178 to −0.3045**	**3.179**	**0.0019**	**0.5069 (0.308 to 0.7375)**
**Peripheral *JUNB* (*mRNA fold change*)**	**0.3417**	**0.1556**	**0.0534 to 0.666**	**2.196**	**0.0281**	**1.407 (1.055 to 1.946)**
**Peripheral *SPHK1* (*mRNA fold change*)**	**−0.3298**	**0.1634**	**−0.7067 to −0.07861**	**2.018**	**0.0356**	**0.7191 (0.4932 to 0.9244)**
**Peripheral *KLF4* (*mRNA fold change*)**	**−5.367**	**0.9761**	**−7.567 to −3.68**	**5.499**	**< 0.0001**	**0.0047 (0.00052 to 0.025)**
**Peripheral *EDN1* (*mRNA fold change*)**	**−0.283**	**0.1131**	**−0.5266 to −0.0834**	**2.502**	**0.0124**	**0.7535 (0.5906 to 0.92)**

Multivariate logistic regression model evaluating the prognostic significance of clinical variables, age (years), Child-Pugh score, AFP (ng/mL), AST (IU/L), ALB (g/dL), and mRNA fold changes of Wnt10A, JUNB, SPHK1, EDN1, and KLF4 in hepatocellular carcinoma (HCC). The model reports coefficients, standard errors (SE), 95% confidence intervals (CI), z-values, p-values, and odds ratios (OR) with corresponding 95% CIs.

Variables such as age, gender, ALT, and AST did not show statistically significant associations with HCC in this model (*P* > 0.05).

### 3.5 Predictive performance of peripheral blood biomarkers for HCC diagnosis

To assess the prognostic potential of candidate biomarkers for hepatocellular carcinoma, ROC curve analyses were conducted for gene expression. Diagnostic metrics, including AUROC, sensitivity, specificity, PPV, NPV, odds ratio (OR), and relative risk (RR), were calculated for gene expression levels (as fold changes), along with AFP, total bilirubin (BIL), and albumin (ALB).

**Fig. 4:**
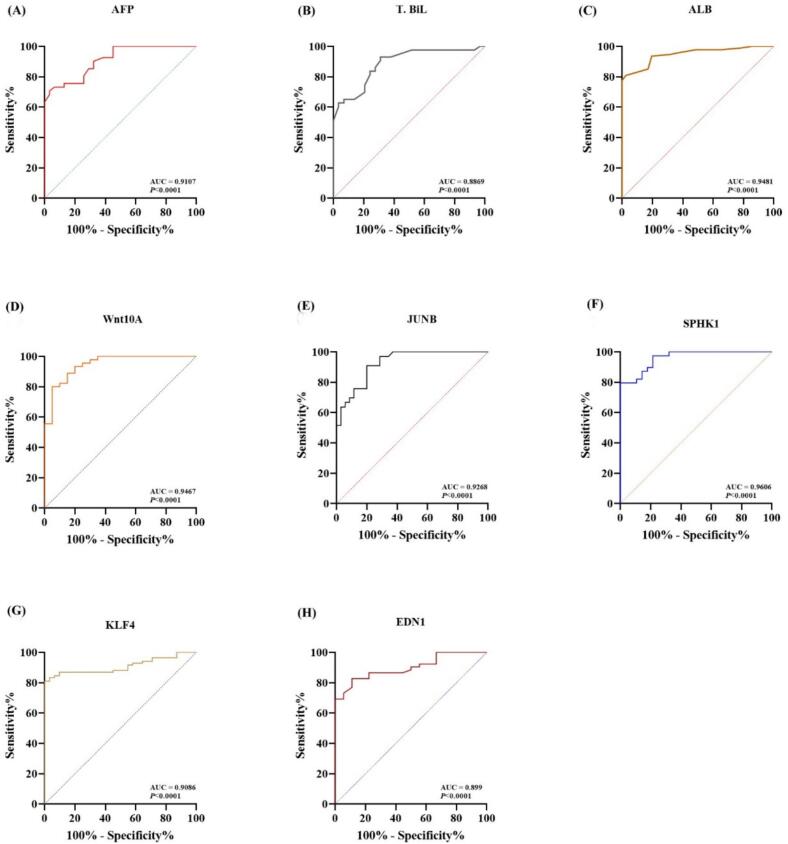
Receiver Operating Characteristic (ROC) Curves for HCC Prognostic Biomarkers ROC curves illustrate the diagnostic performance of five predictor genes (Wnt10A, JUNB, SPHK1, EDN1, and KLF4) as prognostic biomarkers for HCC. The ROC curves collectively demonstrate that all five genes are statistically significant predictors of HCC (P < 0.0001). Optimal cut-off values (>0.9773 for Wnt10A, >2.18 for JUNB, >1.685 for SPHK1, >1.295 for EDN1and < 1.355 for KLF4, > 4.36 for AFP, >4.15 for T.BIL, < 4.15 for ALB) are implied, reflecting thresholds that balance sensitivity and specificity for HCC prognosis yielding the highest prognostic accuracy for HCC. AUC: The area under the curve.

Among the tested biomarkers, SPHK1 demonstrated the highest diagnostic accuracy with an AUROC of 0.9606, sensitivity of 89.74 %, specificity of 82.14 %, and an OR of 59.33. WNT10A closely followed (AUROC 0.9467), with 93.33 % sensitivity, 80.0 % specificity, and OR of 82.67. ALB also showed strong predictive value (AUROC 0.9481), particularly in confirming the absence of HCC, with 97.56 % specificity and OR of 166.3. JUNB (AUROC 0.9268) and KLF4 (AUROC 0.9086) also displayed excellent prognostic performance, with ORs of 111 and 141.8, respectively. AFP, while consistent with its known role, showed lower overall performance (AUROC 0.9107; OR 12.0) compared to the top gene markers. EDN1 recorded an AUROC of 0.899, showing moderate sensitivity but high specificity (94.44 %), while BIL had the lowest AUROC (0.8869), with perfect specificity (100 %) but limited sensitivity (51.16 %). In summary, these results, summarized in Table 6 and Fig. 4, suggest that the expressions of Wnt10A, JUNB, SPHK1, EDN1, and KLF4 genes are promising molecular biomarkers for HCC prognosis, with SPHK1 demonstrating the highest overall discriminatory ability and KLF4 offering the greatest specificity.

**Table 6:** Predictive Cut-Off Values and Diagnostic Performance of HCC Prognostic **Biomarkers.** ROC curve and Fisher’s exact test analysis of candidate genes (*Wnt10A*, *JUNB*, *SPHK1*, *EDN1*, *KLF4*) and classical biomarkers (AFP, ALB, T.BiL) evaluated as prognostic indicators for hepatocellular carcinoma (HCC). Diagnostic metrics include AUROC, sensitivity, specificity, PPV, NPV, odds ratio (OR), and relative risk (RR), with 95% confidence intervals.

Predictor gene		AUROC Test					Fisher’s Exact Test					
	**AUC**	**SE**	**95 % CI** **(*P* value)**	**Cut-off**	**Sensitivity%** **(95 %CI)**	**Specificity%** **(95 %CI)**	**Sensitivity%** **(95 %CI)**	**Specificity%** **(95 %CI)**	**PPV%** **(95 %CI)**	**NPV%** **(95 %CI)**	**Relative risk** **(95 % CI)**	**Odds ratio** **(95 % CI)**
AFP (ng/mL)	0.9107	0.0318	0.8485 to 0.9729 (*P* < 0.0001)	>4.36	80.49 (65.99 to 89.77)	74.19 (56.75 to 86.3)	60.0 (46.18 to 72.39)	88.89 (80.74 to 93.85)	75.0 (59.81 to 85.81)	80.0 (71.12 to 86.66)	3.75 (2.454 to 5.798)	12.0 (4.954 to 29.38)
T. BiL mg/dL)	0.8869	0.0383	0.8119 to 0.9619 (*P* < 0.0001)	>1.27	51.16 (36.75 to 65.38)	100 (88.3 to 100)	49.33 (38.33 to 60.4)	95.38 (87.29 to 98.74)	92.5 (80.14 to 97.42)	62.0 (52.21 to 70.9)	2.434 (1.88 to 3.21)	20.12 (5.881 to 64.48)
ALB (g/dL)	0.9481	0.0173	0.9141 to 0.9821 (*P* < 0.0001)	<4.15	80.85 (71.75 to 87.53)	97.56 (87.4 to 99.87)	67.24 (54.42 to 77.92)	98.78 (93.41 to 99.94)	97.5 (87.12 to 99.87)	81.0 (72.22 to 87.49)	5.132 (3.493 to 7.809)	166.3 (26.89 to 1710)
*Wnt10A* (fold)	0.9467	0.028	0.892 to 1.0 (*P* < 0.0001)	>0.9773	93.33 (82.14 to 97.7)	80.0 (58.4 to 91.93)	88.57 (74.05 to 95.46)	91.43 (84.51 to 95.43)	77.5 (62.5 to 87.68)	96. (90.16 to 98.43)	19.38 (7.74 to 50.08)	82.67 (23.3 to 239.8)
*JUNB* (fold)	0.9268	0.029	0.87 to 0.984 (*P* < 0.0001)	>2.18	90.91 (76.43 to 96.86)	80.0 (64.11 to 89.96)	78.72 (65.1 to 88.01)	96.77 (90.94 to 99.12)	92.5 (80.14 to 97.42)	90.0 (82.56 to 94.48)	9.25 (5.26 to 16.84)	111 (28.33 to 365.7)
*SPHK1* (fold)	0.9606	0.0195	0.9223 to 0.9989 (*P* < 0.0001)	>1.685	89.74 (76.42 to 95.94)	82.14 (64.41 to 92.12)	80 (67.64 to 88.45)	93.68 (86.9 to 97.07)	88 (76.2 to 94.38)	89 (81.37 to 93.75)	8.0 (4.665 to 14.18)	59.33 (20.1 to 176.2)
*KLF4* (fold)	0.9086	0.0272	0.8554 to 0.9618 (*P* < 0.0001)	>1.355	84.52 (75.3 to 90.73)	93.55 (79.28 to 98.85)	82.22 (68.67 to 90.71)	96.84 (91.12 to 99.14)	92.5 (80.14 to 97.42)	92.0 (85.0 to 95.89)	11.56 (6.119 to 22.62)	141.8 (33.77 to 473.5)
*EDN1* (fold)	0.899	0.036	0.8286 to 0.9695	>1.295	73.08 (79.75 to 83.23)	94.44 (74.24 to 99.72)	72.0 (58.33 to 82.53)	95.56 (89.12 to 98.26)	90.0 (76.95 to 96.04)	86.0 (77.86 to 91.47)	6.429 (4.0 to 10.65)	55.29 (17.81 to 154.9)

Gene expression levels are expressed as fold changes. For each gene, the area under the ROC curve (AUROC) is reported with standard error (SE), 95% confidence interval (CI), and p-value. Optimal cut-off values were determined to maximize diagnostic accuracy. Corresponding sensitivity, specificity, positive predictive value (PPV), negative predictive value (NPV), relative risk (RR), and odds ratio (OR) are reported with 95% CIs.

### 3.6 Integrated in-silico epigenetic and pathway analysis

#### 3.6.1 In-silico correlation analysis of histone-modifying enzymes with dysregulated genes in HCC

**Fig. 5:**
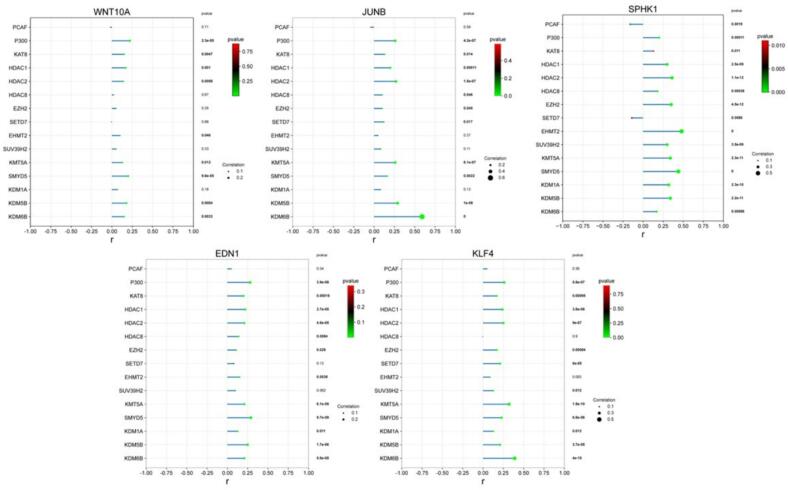
Correlations between KLF4, SPHK1, WNT10A, EDN1, and JUNB expression and histone-modifying enzymes in HCC.

To explore the epigenetic mechanisms driving dysregulation of our target genes in HCC, we examined their correlations with key histone-modifying enzymes using the TCGA-LIHC dataset (GEPIA2) (Fig. 5). These included acetyltransferases (PCAF, P300, KAT8), deacetylases (HDAC1, HDAC2, HDAC8), methyltransferases (EZH2, SETD7, EHMT2, SUV39H2, KMT5A, SMYD5) and demethylases (KDM1A, KDM5B, KDM6B. WNT10A showed modest positive correlations, however the strongest were noted with P300 (r = 0.22, p = 2.3 × 10^−5^), SMYD5 (r = 0.20, p = 9.8 x 10^−5^), HDAC1 (r = 0.17, p = 0.001) and KDM5B (r = 0.18p = 0.0004). JUNB displayed a striking correlation with KDM6B (r = 0.59, p = 0), and additional associations with KDM5B (r = 0.29) and HDAC2 (r = 0.27). SPHK1 exhibited the broadest profile correlating with numerous histone−modifying enzymes. It strongly correlated with EHMT2 (r = 0.48, p = 0), SMYD5 (r = 0.44, p = 0), and HDAC2 (r = 0.36, p = 1.1 × 10^−12^). EDN1 was positively associated with SMYD5 (r = 0.29, p = 9.7 × 10^−9^), P300 (r = 0.28, p = 3.9 × 10^−8^), and KDM5B (r = 0.25, p = 1.7 × 10^−6^). Finally, KLF4 correlated with KDM6B (r = 0.39, p = 4.0 × 10^−15^) and P300 (r = 0.26, p = 5.6 × 10^−7^), as well as HDAC1/2, KMT5A, and SMYD5. Overall, these results suggest that dysregulation of the studied genes is shaped by an active epigenetic network, where multiple histone modifiers converge to influence their expression.

Lollipop plots show Pearson correlation coefficients (x-axis) between the mRNA expression of each gene and histone modifiers (y-axis). Circle size represents correlation strength, and color indicates p-value (red = least significant, green = most significant). Exact p-values are shown on the right side of each plot.

#### 3.6.2 Protein-protein interaction network

**Fig. 6:**
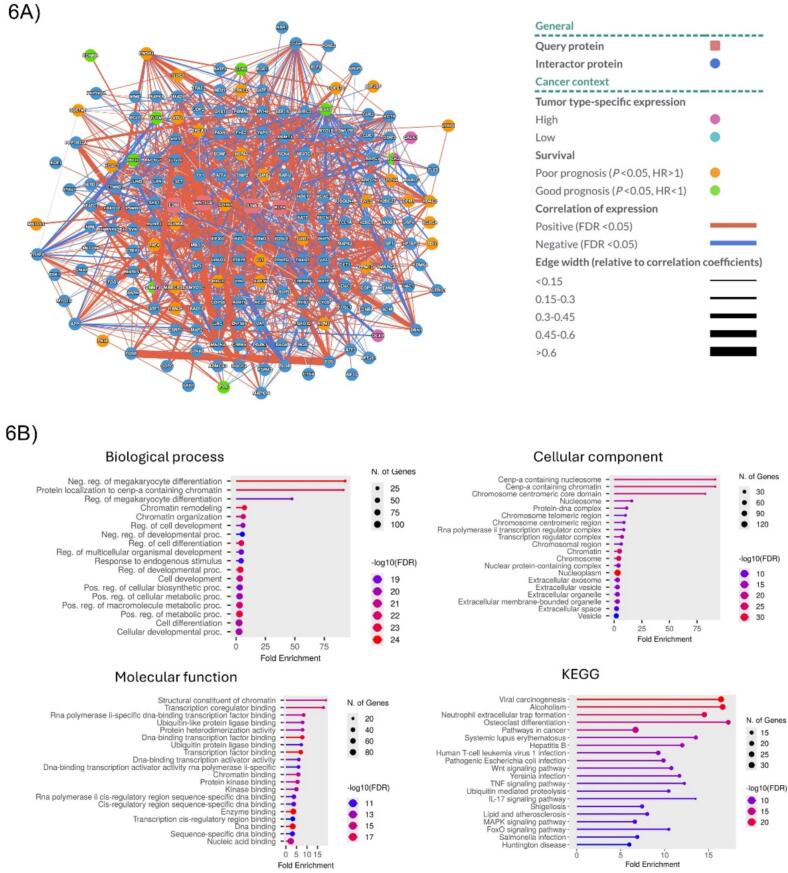
Protein–protein interaction (PPI) network and functional enrichment analyses. 6A) Protein–protein interaction (PPI) network centered on JUNB, EDN1, WNT10A, SPHK1, and KLF4. Circular central nodes represent the studied genes, connected to high-confidence interactors identified by computational analysis. Edges between nodes indicate co-expression correlations, with color denoting direction (red = positive, blue = negative) and width reflecting strength of correlation (r < 0.15 to > 0.6). Node features provide additional context: inner circle color shows tumor type–specific expression (high/low), and outer circle indicates prognostic value (good/poor). 6B) GO and KEGG pathway enrichment of network-associated proteins. GO and KEGG enrichment highlight the most significantly enriched biological terms and pathways. The x-axis shows fold enrichment, dot color indicates FDR-adjusted p-values, and dot size reflects the number of associated genes. (For interpretation of the references to color in this figure legend, the reader is referred to the web version of this article.)

The protein–protein interaction (PPI) network analysis (Fig. 6A) revealed that our five studied proteins do not work separately but are integrated into a highly interconnected regulatory network enriched in epigenetic modulators and transcription factors. The network complexity suggests that dysregulation of these five genes arises not by chance but reflects a coordinated disruption of chromatin and transcriptional regulation. Notably, key interacting proteins include members of chromatin-remodeling complexes and transcription factors, highlighting how perturbation of a single epigenetic regulator, such as an HDAC, could propagate through multiple pathways, driving hepatocarcinogenesis.

#### 3.6.3 Functional enrichment and pathway analyses of the PPI network proteins

Functional enrichment analysis of the PPI network proteins (Fig. 6B) further supported their biological relevance. In Gene Ontology (GO) analysis, the most significantly enriched Biological Processes were chromatin remodeling, chromatin organization, and negative regulation of cell differentiation. Within the Cellular Components category, enrichment of the Cenp-A-containing nucleosomes pointed to their association with fundamental chromatin structures. For Molecular Functions, transcription factor binding and histone-lysine N-methyltransferase activity were among the most prominent terms, indicating roles in transcriptional regulation and histone modification. Complementary KEGG pathway analysis highlighted enrichment in pathways in cancer, Wnt signaling, viral carcinogenesis in hepatitis C, and FoxO signaling. These enrichments indicate that the interacting proteins are linked to epigenetic regulation and oncogenic signaling networks.

## 4 Discussion

Despite global success in hepatitis C virus elimination, hepatocellular carcinoma (HCC) development remains a post-SVR risk, indicating that viral clearance does not fully abolish oncogenesis. Several studies reported that HCV infection triggers gene dysregulation that persists after viral clearance, frequently driven by epigenetic changes.^23^, ^24^ Out of those, we chose a set of genes comprising Wnt10A, JUNB, SPHK1, EDN1, and KLF4. Our in-silico analyses further supported this link by revealing associations between these genes and histone-modifying enzymes, as well as their integration into functionally enriched protein–protein interaction networks and pathways. We focused on analyzing the mRNA expression profiles of these genes in HCC tissues and PBMCs of patients with advanced HCC (HCV-related with and without DAA treatment, and non-HCV-related) compared to chronic HCV patients without malignancies and healthy controls. Our findings confirmed significant differential expressions of these genes in both HCC liver tissues and PBMCs. WNT10A, JUNB, SPHK1, and EDN1 were significantly upregulated and KLF4 downregulated in HCC tissues and PBMCs, suggesting an HCV-driven oncogenic memory. Multivariate regression and ROC curve analyses validated their utility as diagnostic and prognostic biomarkers for HCC, particularly within the DAA-SVR population.

According to HCV and DAA status, expression patterns varied both in liver tissues and PBMCs. In hepatic tissues, WNT10A, JUNB, SPHK1, and EDN1 were consistently elevated in all HCC groups compared to healthy controls, while KLF4 was reduced. Among HCC subgroups, only JUNB and EDN1 showed significant differences: JUNB was markedly higher in untreated HCV-HCC (P < 0.0001), potentially linking active infection to tumor progression, whereas EDN1 was lower in DAA-treated HCC (P < 0.05), suggesting viral clearance may partially modulate its oncogenic activity.

In PBMCs, WNT10A, JUNB, EDN1 and SPHK1 were significantly upregulated, and KLF4 downregulated in chronic HCV and HCV-induced HCC patients, even post-SVR, supporting the concept of an HCV-induced “oncogenic memory.” Although minor differences in gene expression were observed between SVR patients with and without HCC, these were not statistically significant and likely represent background variability rather than a protective effect of DAAs. This is consistent with evidence that SVR, whether after IFN or DAA therapy, reduces but does not eliminate HCC risk, particularly in patients with advanced fibrosis or cirrhosis, who remain at residual risk and require continued surveillance.^5^, ^9^, ^12^ As HCV is thought to promote tumorigenesis indirectly through persistent molecular and epigenetic alterations.^7^ Long-term follow-up is needed to reveal meaningful associations. Indeed, Hamdane et al.^23^ showed that sustained SPHK1 upregulation predicted increased HCC risk over 10 years post-SVR. By contrast, the short-term, minimal, and non-significant changes observed in our cohort are more likely background noise than a protective effect.

In this regard, the debate continues over whether DAAs and interferon (IFN)-based regimens differ in their long-term impact on HCC risk. While initial studies suggested higher rates of HCC occurrence after DAA therapy^37^, ^38^, larger and more recent analyses have not supported this finding.^6^, ^12^ Since IFN therapy is now rarely used and DAAs have become the standard of care, most contemporary cohorts, including ours, are predominantly composed of DAA-treated patients.

In addition to expression profiling we conducted in-silico analyses to investigate the epigenetic landscape underlying the dysregulation of our candidate genes. The enrichment of chromatin remodeling, histone-modifying activity, and transcription factor binding within the PPI network suggests that dysregulation of our candidate genes reflects a wider disturbance in chromatin architecture rather than isolated transcriptional events. KEGG pathway analysis further linked the network to Wnt signaling, FoxO signaling, and viral carcinogenesis, reinforcing their relevance to HCV-driven HCC. WNT10A’s role in Wnt/β-catenin signaling, JUNB’s integration with AP-1 pathways, SPHK1′s involvement in immune evasion, EDN1′s angiogenic and proliferative functions, and KLF4′s regulation within FoxO signaling illustrate how these genes converge on key oncogenic and epigenetic mechanisms. Collectively, these findings support the view that their persistent dysregulation is embedded within broader signaling and epigenetic networks contributing to hepatocarcinogenesis.

The elevated expression of WNT10A (7.11-fold) in PBMCs of patients with chronic HCV infection remained high in HCV-induced HCC cases, independent of antiviral treatment (2.04-fold in the DAAs cured HCV-HCC group, 2.17-fold in the SVR group, and 2.69-fold in the non-treated HCV-HCC group). These findings underscore WNT10A’s role in Wnt/β-catenin signaling, driving proliferation, EMT, and metastasis, thereby linking chronic inflammation and viral infection to hepatocarcinogenesis.^39^, ^40^ Our results align with prior gene expression profiling showing WNT10A upregulation in PBMCs of HCV-related HCC patients^41^, and a transcriptomic study showed that WNT10A remained epigenetically upregulated in HCV-infected liver tissue even after DAA-mediated viral clearance.^24^

Similarly, JUNB was markedly upregulated in PBMCs (2.72-fold in chronic HCV, 2.69-fold in HCV-HCC (no DAAs), 2.06-fold in SVR-HCC, and 1.79-fold in SVR patients). JUNB, a component of the AP-1 transcription factor, exerts context-dependent roles in cancer, acting as a suppressor in some tumors but predominantly as an oncogene in HCC^42^, where it promotes proliferation, angiogenesis, and EMT through AP-1 and TGF-β signaling^42^, ^43^, ^44^, ^45^. Our findings align with previous studies reporting JUNB overexpression in PBMCs of HCC patients, where it correlated with poor prognosis and reduced survival.^46^, ^47^ Its high expression in PBMCs has been linked to immune evasion mechanisms by modulating the tumor immune microenvironment (TME) and impairing NK cell function through interactions with immune-related genes like ANXA2 and S100A4^43^, ^46^, ^47^. Moreover, epigenetic modifications, such as DNA methylation, have also been reported to further sustain JUNB expression by stabilizing its mRNA, reinforcing its role in EMT and tumorigenesis.^24^, ^48^

SPHK1, generating sphingosine-1-phosphate (S1P), was also significantly upregulated in PBMCs of chronic HCV patients (4.84-fold) and persisted in HCC patients despite DAA treatment (1.59-fold in HCV-HCC no DAAs, 1.95-fold in SVR-HCC, and 1.91-fold in SVR patients). Our results align with recent studies demonstrating that SPHK1 expression remains elevated in HCV-infected liver tissue even after successful antiviral therapy, indicating a persistent metabolomic signature of chronic infection. This sustained upregulation of SPHK1, along with elevated S1P levels, has been implicated in immune evasion through PD-L1–mediated T cell exhaustion in chronic HCV infection and to HCC progression post-SVR.^23^, ^49^, ^50^ Notably, Hamdane et al. reported that high SPHK1 expression has also been associated with increased HCC risk up to 10 years post-SVR^23^, while other studies reported significantly higher serum S1P levels in HCC patients compared with cirrhosis or healthy controls, highlighting their potential as diagnostic markers for HCC.^50^, ^51^

Moreover, our findings support the role of EDN1 as a key mediator in HCV-associated HCC, highlighting its contribution to inflammation-driven tumorigenesis. In our study, EDN1 showed marked upregulation in chronic HCV cases (3.96-fold, 1.83–2.08 fold in HCC groups and 2.15-fold in the SVR group), consistent with prior studies linking it to the CXCL8–SRC oncogenic axis in prolonged HCV infection underscoring its role in linking inflammation to tumor initiation.^52^ EDN1 also contributes to fibrosis and promotes angiogenesis, proliferation, migration, and survival through EMT and Wnt/β-catenin pathways^53^, ^54^. Its expression is further induced by VEGF^55^ and histone modifications during HCV-related carcinogenesis.^24^

Conversely, KLF4, was consistently downregulated in PBMCs across all HCC groups (0.24–0.31-fold in HCV-HCC, 0.25-fold in SVR-HCC). KLF4, a zinc finger transcription factor with context-dependent roles in cancer, is predominantly tumor-suppressive in HCC, where its low expression correlates with poor differentiation, unfavorable prognosis, and worse outcomes after liver transplantation.^56^, ^57^ Functionally, it suppresses HCC progression by inhibiting EMT through downregulation of Slug (SNAI2),^58^, ^59^ suppressing EGFR and JNK signaling, reduces migration through inducing P-cadherin.^60^ While some studies suggest oncogenic effects, most evidence supports a tumor-suppressive role, reinforcing its potential as a prognostic biomarker in HCC.^61^, ^62^, ^63^

Overall, the persistence of these gene expression changes in PBMCs after viral clearance highlights their potential as non-invasive biomarkers for monitoring residual HCC risk. Given their role in immune surveillance and tumor interaction, PBMCs provide a practical alternative to liver biopsies, reflecting systemic immune dysregulation and oncogenic activity. Their expression profiles may capture circulating tumor signals or immune responses, supporting their utility in early detection, prognosis, and personalized care in post-SVR HCV-associated HCC.^30^, ^31^, ^64^, ^65^

ROC curve analyses demonstrated robust diagnostic accuracy of the studied genes. SPHK1 showed the highest AUROC (0.96), followed by WNT10A and JUNB (>0.92), while KLF4 showed the highest specificity (93.55 %) across molecular markers, surpassing the diagnostic accuracy of AFP (sensitivity 80.49 %, specificity 74.19 %). Moreover, among classical markers, albumin (ALB) had the highest specificity (97.56 %) but lower sensitivity (77.53 %), supporting its role in confirming the absence of disease rather than early detection.

Multivariate regression identified peripheral SPHK1, WNT10A, EDN1, and KLF4 as inversely associated with HCC risk, while JUNB showed a positive correlation, independent of confounders like age, gender, and liver function markers.^66^, ^67^ These findings highlight the superior discriminatory power of molecular biomarkers compared to AFP, which is elevated in only 60–70 % of HCC cases, and is particularly unreliable in smaller tumors, and DAA-SVR patients who may exhibit normal serum levels despite thrombocytopenia and elevated INR.^68^, ^69^, ^70^

The Clinical characteristics of our cohorts align with the known features of liver carcinogenesis, showing male predominance, advanced age, reduced platelet and albumin levels, and elevated INR and AFP in HCV-related (with or without DAA) and non-HCV-related HCC compared to healthy and chronic HCV patients. All HCC groups exhibited severe liver damage, with higher Child-Pugh scores, lower albumin, and reduced platelet counts compared to non-malignant groups. Notably, AFP levels were lower in SVR patients than in treatment-naïve chronic HCV patients, highlighting the limitations of AFP and reinforcing the need for more accurate molecular markers to enable early diagnosis and risk stratification.^71^, ^72^

Mechanistically, the five studied genes converge on oncogenic processes including Wnt/β-catenin, immune evasion, angiogenesis, and fibrosis. In silico analyses further indicated that these genes are embedded within broader epigenetic and signaling networks, supporting the concept that their persistent dysregulation in PBMCs post-SVR reflects an HCV-driven oncogenic memory. Clinically, these biomarkers may enhance HCC risk stratification in DAA-SVR patients, where severe liver damage and male predominance and the limited reliability of AFP remain challenging.^71^, ^72^

The strengths of our study include its dual liver tissue and PBMC analysis, capturing local and systemic changes, and the complementary in silico validation. However, limitations include modest cohort size and heterogeneous HCC etiologies, and a mean age difference between controls and HCC patients, which we addressed by including age as a covariate in multivariate analyses. In addition, while our bioinformatics analyses provide insight into the epigenetic context of the dysregulated genes, future integration of direct epigenetic profiling (e.g., DNA methylation and histone modifications) will further clarify their mechanistic role.

In conclusion, SPHK1, WNT10A, JUNB, EDN1, and KLF4 emerge as promising non-invasive biomarkers for HCC in DAA-SVR patients. Their persistent dysregulation in PBMCs, reinforced by in-silico evidence of integration into oncogenic and epigenetic networks, highlights their potential for early risk assessment and therapeutic targeting, while future larger and multi-omic studies will be essential to validate and extend these findings.

## CRediT authorship contribution statement

**Rehab I. Moustafa:** Conceptualization, Data curation, Funding acquisition, Methodology, Project administration, Resources, Supervision, Validation, Writing – original draft, Writing – review & editing. **Sally Farouk:** Writing – review & editing, Project administration, Methodology, Formal analysis, Conceptualization. **Noha G. Bader El Din:** Writing – review & editing, Validation, Formal analysis, Data curation, Conceptualization. **Hend I. Shousha:** Conceptualization, Investigation, Resources, Methodology. **Ahmed Khairy:** Validation, Methodology, Investigation. **Yasser K. Elesnawy:** Methodology, Investigation. **Heba Shawky:** Visualization, Validation, Formal analysis, Data curation. **Ahmed M. Gabr:** Investigation, Methodology. **Ashraf O. Abdelaziz:** Supervision. **Amr Abdelaal:** Supervision. **Hassan Elsayed:** Writing – original draft, Visualization, Validation, Methodology, Formal analysis, Data curation, Conceptualization.

## Declaration of competing interest

The authors declare that they have no known competing financial interests or personal relationships that could have appeared to influence the work reported in this paper.
